# Demokratisch und emanzipatorisch – Partizipative Gesundheitsforschung hat hohes Potenzial

**DOI:** 10.1007/s00103-020-03276-8

**Published:** 2021-01-08

**Authors:** Gesine Bär, Claudia Hövener, Michael T. Wright, Anke-Christine Saß

**Affiliations:** 1 Alice Salomon Hochschule Berlin, Alice-Salomon-Platz 5, 12627 Berlin, Deutschland; 2 Abteilung für Epidemiologie und Gesundheitsmonitoring, Robert Koch-Institut, General-Pape-Str. 62–66, 12101 Berlin, Deutschland; 3 Katholische Hochschule für Sozialwesen Berlin, Köpenicker Allee 39–57, 10318 Berlin, Deutschland

Partizipative Gesundheitsforschung ist die aktive Einbeziehung von Praktiker*innen und jenen Menschen, deren Lebensbereiche erforscht werden, in den Forschungsprozess. Das bedeutet auch eine Teilung von Entscheidungsmacht im Forschungsprozess zwischen Wissenschaft, Praxis und Betroffenen. Dies ermöglicht, Themen wie gesundheitliche Ungleichheit, Ausgrenzung und Marginalisierung konsequent aus Sicht derjenigen zu erforschen, die Lebenserfahrungen in diesen Bereichen haben. Entsprechend kann ein emanzipatorischer Nutzen von dieser Forschung erwartet werden, auch wenn sie ressourcen- und zeitaufwändig ist. Sowohl die Daten der Gesundheits- und Sozialberichterstattung [1, 2] rechtfertigen diese Schwerpunktsetzung als auch die Tatsache, dass Menschen, die am vulnerabelsten in Bezug auf gesundheitsgefährdende Bedingungen sind, mit anderen Forschungsansätzen schwer erreicht werden. Folglich werden deren Gesundheitsprobleme und Bedarfe seltener untersucht.

Partizipative Forschung beinhaltet, strukturelle Veränderungen mithilfe forschender Verfahren zu initiieren, zu begleiten, weiterzuentwickeln oder zu evaluieren. Im Ergebnis werden idealerweise Gesundheitsprobleme gelindert oder verhindert, Ressourcen gestärkt sowie die Kapazitäten und Resilienzen auch auf Community- bzw. Gemeindeebene gefördert [3]. Auch Ansätze der partizipativen Epidemiologie versuchen hier neue Wege zu gehen [4]. Der Nutzen partizipativer Forschung für alle Beteiligten ist entsprechend vielseitig: So können Forschende z. B. die Lebenswelt der Forschungsteilnehmenden durch deren Einbeziehung besser verstehen, alle Beteiligten können eigene Anliegen einbringen und direkt an der Planung und Umsetzung relevanter Interventionen mitwirken [5]. Auf verschiedenen Ebenen von Wissenschaft, Politik und Verwaltung, in verschiedenen Institutionen und Settings sowie bei den direkt Beteiligten zeigen sich Wirkungen der Projekte (siehe Beitrag von Allweiss et al. in diesem Themenheft).

Die Rahmenbedingungen partizipativer Forschung haben sich in den letzten Jahren deutlich weiterentwickelt, so wie 2013 im Schwerpunktheft „Partizipative Gesundheitsforschung“ [6] prognostiziert. Gleichwohl kann noch nicht davon gesprochen werden, dass die erwartete Konsolidierungsphase erreicht wäre, wie auch eine jüngere Recherche zeigt [7]. Dennoch wird das Thema „Partizipation in der Forschung“ in Deutschland inzwischen politisch breit diskutiert und findet sich in diversen Förderlinien wieder.^1^ Weitere Beispiele sind die Aufnahme des Kriteriums der Partizipation in das Rahmenprogramm Gesundheitsforschung und die für das Jahr 2022 geplante Partizipationsinitiative des Bundesministeriums für Bildung und Forschung (BMBF). Das Netzwerk Partizipative Gesundheitsforschung (PartNet) hat dies zum Anlass genommen, eine Reihe von Förder- und Strukturanforderungen zu formulieren (vgl. [8] sowie Bethmann et al. in diesem Heft).

Im vorliegenden Schwerpunktheft werden die Potenziale Partizipativer Gesundheitsforschung aufgezeigt. Die Beiträge entstammen überwiegend Projekten, die vom BMBF gefördert wurden. Zentral für die BMBF-geförderte Partizipative Gesundheitsforschung war der Forschungsverbund PartKommPlus, aber auch Verbünde wie Capital4Health oder AEQUIPA hatten partizipative Anteile. Die Auswahl der Beiträge verdeutlicht die Vielfalt der geförderten partizipativen Verbundforschung. Auffallend ist hierbei die besondere Rolle von Hochschulen für angewandte Wissenschaften und von praxisnahen Forschungseinrichtungen. Entsprechend findet sich unter den hier vertretenen Autor*innen eine Reihe von Praxispartner*innen, was die enge Zusammenarbeit bei der Ergebnisdarstellung zeigt.

Im einleitenden Beitrag führt Wright aus, dass sich Partizipative Gesundheitsforschung national wie international in einer Phase dynamischer Weiterentwicklung befindet. Aktuelle Themen werden benannt. Interessant ist der Hinweis, dass die Ansätze von Gemeinschafts- und Praxisforschung zwar unterschiedlich sind, aber häufig in Kombination auftreten.

Der methodologische Beitrag von Kasberg et al. definiert den Methodenbegriff für die Partizipative Gesundheitsforschung und schlägt auf der Basis eines Scoping-Reviews eine Systematisierung vor. Methoden der partizipativen Prozessgestaltung wird neben den Forschungsmethoden ein wichtiger Stellenwert eingeräumt.

Die Partizipative Gesundheitsforschung macht den Forschungsprozess selbst zum Gegenstand von Erkenntnisgewinn und sozialer Intervention und verknüpft somit Erkenntnisgewinn und konkreten Handlungsbezug. Dies bildet sich sehr deutlich in den Beiträgen dieses Heftes zu Rollenverteilungen im Forschungsprozess (Kümpers et al.), zum Gewinnen von „selten Gehörten“ für die partizipative Forschung (Schaefer et al.) und zu ethischen Reflexionen (Schaefer & Narimani) ab. Die Bedeutung von Beziehungs- und Kooperationsaufbau, die gemeinsame Prozessreflexion und schließlich die Erweiterung von Handlungsspielräumen werden in allen drei Beiträgen bilanziert.

Die Anwendung partizipativer Ansätze ist vielfältig und orientiert sich sehr am Kontext der Beteiligten und des Gesundheitsthemas, das im Vordergrund steht. Einige Beiträge gehen auf die praktische Umsetzung von Partizipativer Gesundheitsforschung in unterschiedlichen Bereichen ein, wie in der Offenen Kinder- und Jugendarbeit (Rataj et al.), der Bewegungsförderung (Gelius et al.) und den integrierten kommunalen Strategien für Gesundheitsförderung (Wihofszky et al.). Dabei wird ein interessantes Spektrum methodischer Ansätze deutlich: partizipative Evaluation, kooperative Planung und eine interaktive Standortanalyse.

Die letzten Beiträge des Hefts deuten auf Besonderheiten der Partizipativen Gesundheitsforschung hin. Hilgenböcker et al. heben das Potenzial von nachhaltiger Qualitätsentwicklung durch die Beteiligung von Bürger*innen und lokalen Verantwortlichen an Methoden der Datenerhebung, -auswertung und -aufbereitung für die Praxis hervor. Allweiss et al. setzen sich mit der Frage auseinander, wie der Mehrwert der Partizipativen Gesundheitsforschung erfasst und dargestellt werden kann. Dabei nehmen sie Bezug auf die internationale Diskussion zum gesellschaftlichen Nutzen der Forschung über die Erzeugung wissenschaftlicher Erkenntnisse hinaus. Bethmann et al. thematisieren aus Sicht von Forschenden die spezifischen Förder- und Rahmenbedingungen, die Partizipative Gesundheitsforschung erfordert.

Die veränderten Rahmenbedingungen wie die Impulse aus den Forschungsprojekten stimmen optimistisch, dass sich das Feld der Partizipativen Gesundheitsforschung weiter etablieren wird. Die Beiträge haben aber auch unterstrichen, dass viele Debatten erst begonnen haben und noch intensiverer Bearbeitung bedürfen. Partizipative Gesundheitsforschung erscheint aufgrund der beteiligten Vielfalt von Akteur*innen und der Integration von lebensweltlichem, berufspraktischem und wissenschaftlichem Wissen ressourcenaufwändig und herausfordernd. Ihr demokratisches und emanzipatorisches Potenzial macht sie attraktiv. Die Erträge partizipativer Verfahren in Bezug auf ihre Aussagekraft und Relevanz für alle Beteiligten klar herauszuarbeiten bleibt eine kontinuierliche und wichtige Zukunftsaufgabe.

## Footnotes

1 Kleine Anfrage zu „Bürgerwissenschaft und partizipativer Forschung“ von 2018 (Drucksache des Deutschen Bundestages 19/362519).
